# Penetrating Ocular Fish-Hook Injury

**DOI:** 10.7759/cureus.48872

**Published:** 2023-11-15

**Authors:** Mustafa Hammad, Husain Alharmi, Abdulhameed H Mahmood

**Affiliations:** 1 General Surgery, West Suffolk Hospital, Bury St Edmunds, GBR; 2 General Surgery, Royal College of Surgeons in Ireland - Bahrain, Muharraq, BHR; 3 Ophthalmology, Salmaniya Medical Complex, Manama, BHR

**Keywords:** fish hook removal from eye, fishhook eye injury, fishhook in anterior chamber, eye foreign body, penetrating eye injury, protective eyewear, penetrating ocular injuries, fish hook

## Abstract

Penetrating ocular injuries caused by fish hooks, although rare, present unique challenges and significant risks to ocular structures and vision. We report a case of a 36-year-old male who presented with a fish hook embedded in his right eye. Clinical examination revealed a fish hook perforating the cornea, entering at the nasal cornea at 3 o'clock and exiting at the temporal cornea at 7 o'clock. Despite initial attempts to employ the advance-and-cut technique, the unsuccessful utilization of a wire cutter led to a shift in the removal approach. The modified backout method was successfully employed, allowing the safe extraction of the fish hook while minimizing iatrogenic damage. Follow-up appointments showed a gradual improvement in visual acuity despite early cataract formation and significant central scarring. To optimize the patient's visual outcome, a triple procedure involving penetrating keratoplasty (PKP), extra-capsular cataract extraction (ECCE), and intraocular lens (IOL) implantation has been scheduled. This case highlights the importance of prompt and adaptable management in ocular fish-hook injuries, emphasizing the need for comprehensive follow-up care. It also underscores the value of preventive measures, including the use of protective eyewear, to reduce the incidence of such injuries, given that the majority of ocular traumas are preventable.

## Introduction

Accidents involving the eye can have significant consequences on vision, and injuries caused by fish hooks penetrating the eye are particularly severe. While rare, these incidents can result in damage to multiple ocular and surrounding structures, which can lead to visual morbidity and complications such as infection, intraocular hemorrhage, traumatic cataract, corneal scarring, and retinal detachment [1,2]. We present the details of the successful removal of a penetrating ocular fish hook utilizing the modified backout method following a failed attempt of the advance-and-cut technique, two of several techniques documented in the literature to safely remove fish hooks from the eye [3]. This case report emphasizes the importance of timely medical intervention and follow-up to optimize patient outcomes. It also aims to raise awareness about the risks associated with activities involving sharp objects, highlighting the importance of complying with recommended safety measures. This is especially crucial, considering that an estimated 90% of ocular trauma is preventable [4].

## Case presentation

**Figure 1:**
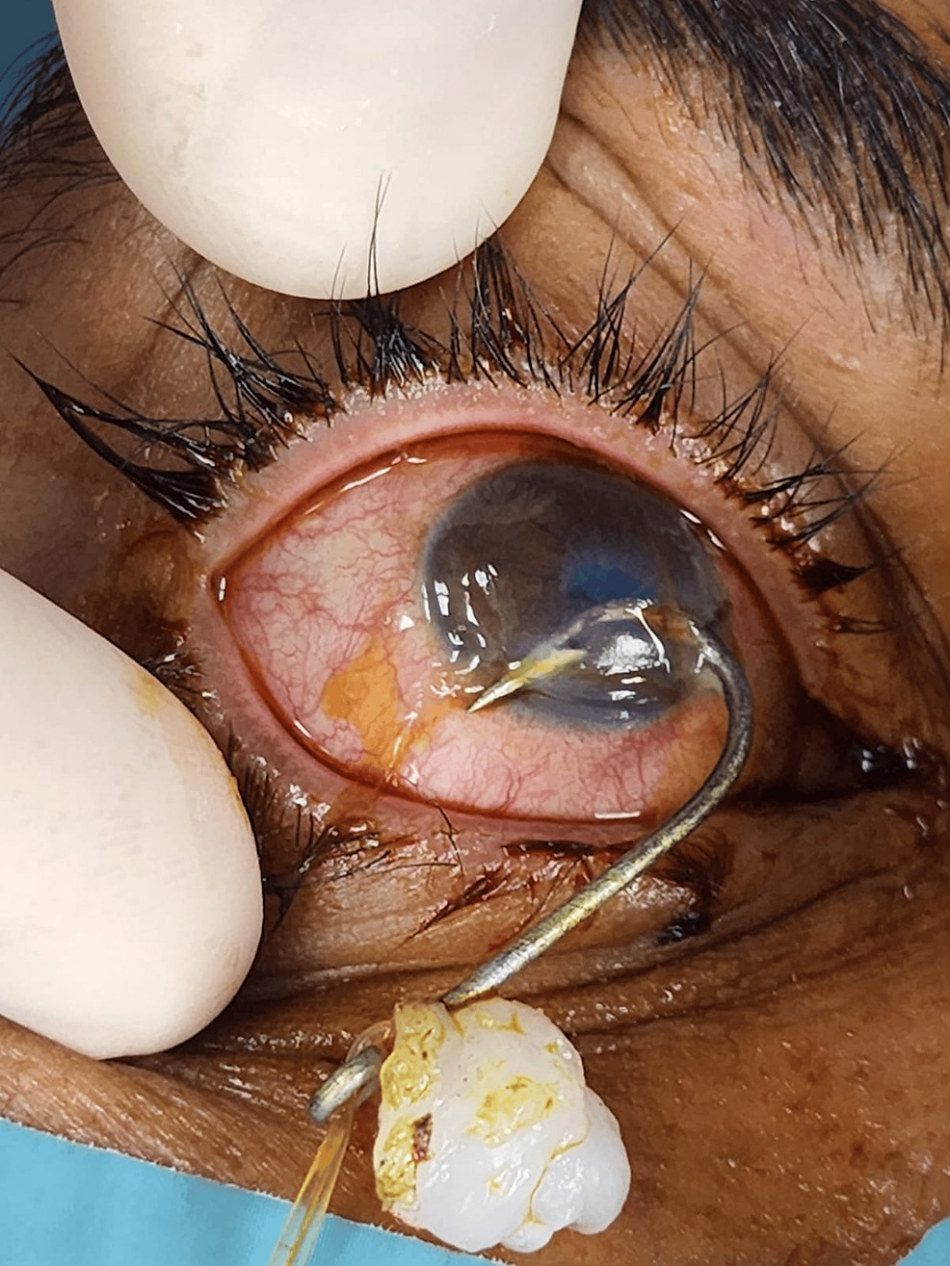
Large Fish Hook Penetrating the Anterior Chamber of the Eye With Entry and Exit Wounds at 3 o'clock and 7 o'clock, Respectively

**Figure 2:**
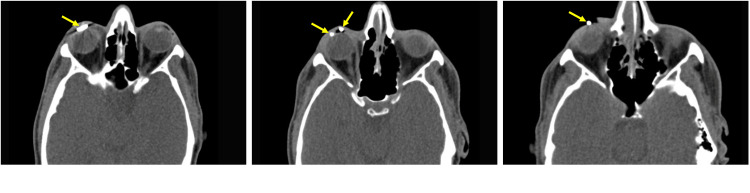
Transverse CT Head Series Showing Fish Hook Lodged in the Anterior Chamber of the Eye Yellow arrows indicate the fish hook in the anterior chamber of the eye, represented as a white opacity.

**Figure 3:**
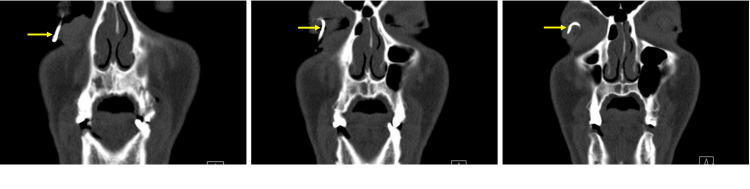
Coronal CT Head Series Showing Sequential Progression of the Fish Hook's Position in the Anterior Chamber of the Eye Yellow arrows denote the progression of the fish hook within the anterior chamber of the eye, represented as a white opacity.

A 36-year-old male presented to the emergency department with a fish hook embedded in his right eye, a distressing ocular injury. Upon examination of the affected eye, the patient displayed normal lid anatomy, congested conjunctiva, and a fish hook penetrating the cornea, entering at the nasal cornea at 3 o'clock and exiting at the temporal cornea at 7 o'clock (Figure 1). The clinical evaluation revealed a visual acuity limited to counting fingers at close proximity and a shallow anterior chamber with an inaccessible view of the fundus. Computed tomography (CT) of the orbits confirmed the presence of the fish hook within the anterior chamber, with no radiopaque intraocular foreign body detected in the vitreous cavity (Figure 2). The sequential progression of the fish hook's position within the anterior chamber is illustrated in the coronal CT head series (Figure 3). It is noteworthy that the patient reported not wearing protective eyewear at the time of the incident. 

**Figure 4:**
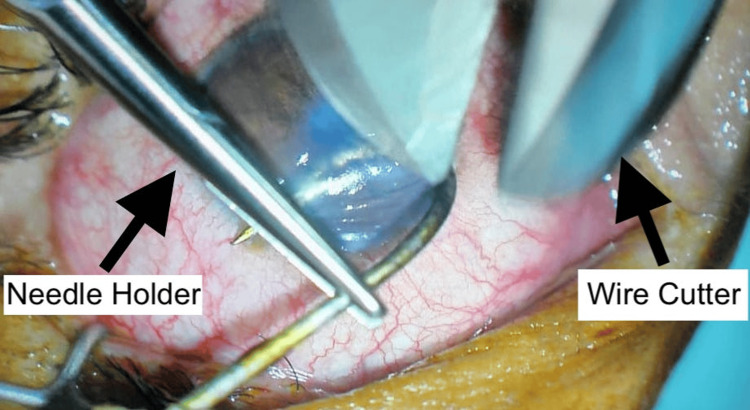
Unsuccessful Wire Cutter Use in Advance-and-Cut Technique

In consideration of the patient's condition, a decision was made to proceed with the removal of the fish hook and primary corneal repair under general anesthesia. The initial approach involved an attempt to employ the advance-and-cut technique, recognized as the preferred method for managing barbed hooks. However, the endeavor to cut the fish hook with a wire cutter proved unsuccessful (Figure 4).

Subsequently, the modified backout method was implemented as a highly effective alternative. This method entailed the cautious withdrawal of the fish hook through the entry wound, involving the enlargement of the entry site to create a secure pathway for the barb's safe passage, thereby minimizing the risk of additional iatrogenic trauma. The fish hook was meticulously extracted using a combination of forceps and a needle holder. To ensure wound closure, nine 10-0 nylon sutures were meticulously placed. A bimanual anterior chamber washout was performed, and intracameral moxifloxacin, along with subconjunctival gentamicin and dexamethasone, were administered.

Postoperatively, the patient received tetanus toxoid vaccine and intravenous cefuroxime, accompanied by topical antibiotics (moxifloxacin) and topical steroids. A bandage-contact lens was placed to facilitate corneal healing. The patient was discharged on the following day, with instructions to follow up with the corneal specialist for further evaluation and care.

Subsequent follow-up appointments revealed a progressive improvement in the visual acuity of the patient's right eye. At the one-month follow-up, there was an improvement in visual acuity from a baseline of counting fingers near the face to the ability to count fingers at a distance of 3 meters. During this evaluation, one suture was thoughtfully removed due to its interference with the visual axis. Upon the six-month follow-up assessment, all remaining sutures were removed as part of the ongoing management plan. Despite the persistence of an early cataractous lens and the presence of substantial central scarring, the patient's visual acuity exhibited substantial improvement. Notably, the patient achieved a visual acuity of 6/60 without correction, which further improved to 6/18 with the use of a pinhole occluder. With the aim of optimizing the patient's visual outcome, a decision was made to proceed with a triple procedure, which will encompass penetrating keratoplasty (PKP), extra-capsular cataract extraction (ECCE), and intraocular lens (IOL) implantation.

## Discussion

**Figure 5:**
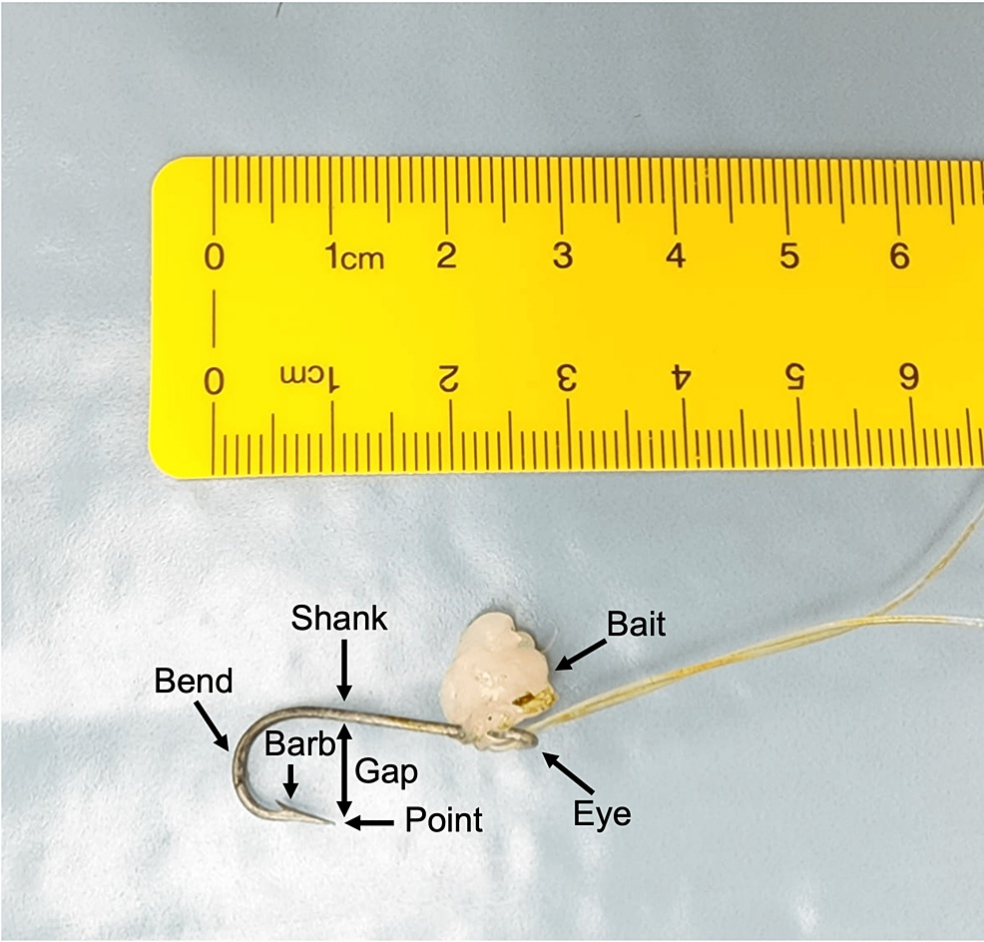
Anatomy of a Fish Hook

There is an expanding body of literature concerning ocular fish hook injuries. Such injuries have been documented in prior case reports and their management demands a planned approach that considers factors such as the hook's anatomy, size, and barb characteristics (Figure 5) [5].

Selecting the appropriate removal technique is contingent upon a careful evaluation of these details to minimize additional damage to the eye and surrounding structures. Among the methods recommended in the literature, the advance-and-cut technique is frequently favored, particularly for anterior segment fish hook injuries. In this technique, the point and barb of the fish hook are advanced through the wound until they emerge from the eye, subsequently allowing the fish hook to be grasped and transected using a wire cutter. This renders the barbless hook easily removable from the entry wound [6]. In our case, the initial attempt to employ the advance-and-cut method was hindered by the unavailability of an appropriate wire cutter, emphasizing a potential drawback to this technique. Notably, the method's success is reliant on the availability of specialized equipment, and its execution may exert excessive strain on the eye during the cutting process.

As an alternative to the advance-and-cut technique, we adopted the modified backout method. The standard backout method entails retrograde removal of the fish hook through the entry wound. While this method is typically suitable for barbless fish hooks, the presence of a barb can result in excessive damage to ocular tissues [1]. The modified version of this technique, as applied in our case, involves creating an entry wound incision adequate for the barb's extraction without impinging upon ocular structures [7,8].

Recent innovations in fish hook removal techniques have included a modification of the advance-and-cut method that obviates the need for a wire cutter. This variation involves advancing the point and barb through the wound until they protrude from the eye, after which the barb is compressed and bent over the body of the bend using a needle holder. This renders the hook barbless and facilitates its safe retrograde removal from the entry site [9].

In the context of ocular injuries, follow-up care is of paramount importance for determining the prognosis of visual acuity. As illustrated in this case, the patient developed traumatic cataract and corneal scarring, both of which can significantly impact vision. These complications are amenable to treatment, and scheduling the patient for a triple procedure can notably enhance visual outcomes.

## Conclusions

Ophthalmologists must be well-prepared to address the unique clinical and surgical challenges associated with intraocular fish hook injuries. When managed promptly and effectively, these injuries often yield favorable long-term prognoses. Furthermore, raising awareness and actively promoting preventive measures, such as advocating for the use of protective eyewear by both fishermen and observers, is pivotal. By doing so, we can make substantial contributions to reducing the incidence and severity of these accidents given that most cases of ocular trauma are preventable.
